# Modification of Apple Pomace by Extrusion Processing: Studies on the Composition, Polymer Structures, and Functional Properties

**DOI:** 10.3390/foods9101385

**Published:** 2020-10-01

**Authors:** Vera Schmid, Antje Trabert, Judith Schäfer, Mirko Bunzel, Heike P. Karbstein, M. Azad Emin

**Affiliations:** 1 Institute of Process Engineering in Life Sciences, Chair of Food Process Engineering, Karlsruhe Institute of Technology (KIT), 76131 Karlsruhe, Germany; vera.schmid@kit.edu (V.S.); heike.karbstein@kit.edu (H.P.K.); 2 Institute of Applied Biosciences, Department of Food Chemistry and Phytochemistry, Karlsruhe Institute of Technology (KIT), 76131 Karlsruhe, Germany; antje.trabert@kit.edu (A.T.); judith.schaefer@kit.edu (J.S.); mirko.bunzel@kit.edu (M.B.)

**Keywords:** upcycling, valorization, by-products, dietary fiber, plant cell wall, non-starch polysaccharides, rheology

## Abstract

By-products of fruit and vegetable processing are an inexpensive and sustainable source of dietary fiber, potentially offering valuable functional properties such as water binding and thickening. Due to these favorable properties, they can be utilized to reformulate widely-consumed foods, e.g., bakery products or beverages. In this study, apple pomace was used as a model system to study whether extrusion technology affects food by-product functionality and thus has the potential to broaden the application of by-products in foods. The effect of the process parameters and the extent of thermo-mechanical treatment on the structural and functional properties of apple pomace were analyzed after extrusion trials using various screw speeds, water contents, and barrel temperatures. Compared to the raw material, apple pomace extruded at *T*_barrel_ = 100 °C, *n* = 700 min^−1^ and *m*_H2O_ = 17% showed an increased water solubility up to 33%. The water absorption increased from 5 to 19 Pa·s and the paste viscosity from 5 to 339 Pa·s by extrusion processing. Analyses of dietary fiber contents and fiber polysaccharide structures revealed that thermo-mechanical stress (*n* = 700 min^−1^, *m*_H2O_ = 22%) increased the content of soluble dietary fiber from 12.5 to 16.7 g/100 g dry matter, and that the harshest conditions even enabled the formation of low-molecular-weight dietary fiber. Arabinans (as neutral rhamnogalacturonan I side chains) appeared to be most sensitive to thermo-mechanical stress, whereas xylans (i.e., a group of minor polysaccharides) were an example of a more stable fiber polysaccharide. Also, the degree of methylation of the pectic polysaccharides was strongly reduced from 50% to 15% when thermo-mechanical stress was applied. Imaging and pore size analysis showed that extrusion processing could disrupt the rigid cell wall macromolecular structure.

## 1. Introduction

Lately, by-products of fruit and vegetable processing have been attracting increasing attention in the fields of food research and industry as affordable, sustainable, and natural sources of both dietary fiber [1,2] and low-molecular-weight bioactive compounds [1,3,4]. Furthermore, they show a wide variety of techno-functional properties, such as water- and oil-absorbing and thickening and gelling properties [5,6,7]. By partially replacing synthetic food additives, they meet the demand of consumers for more natural and sustainable food products [6,8,9,10]. The incorporation of fibers can, therefore, impart favorable properties upon foods such as baked goods, dairy products, sausages, and beverages. However, the main challenge is that unprocessed by-products commonly have no or low functionality, which limits their application. Since they often unfavorably affect the sensory and/or textural characteristics of food products, they can be added to food systems in limited concentrations only [5,11,12,13,14,15,16,17]. To increase the amount of dietary fiber without having a negative impact on product’s properties, and to utilize the full potential of by-products, significant research is being undertaken on the modification of functional properties, e.g., water absorption and gelling of dietary fiber by various methods such as chemical, enzymatic, thermal, and mechanical treatment [6,14,18,19]. Extrusion processing has several advantages as an efficient and continuous process with high flexibility, allowing its application to a wide range of by-products. Modification is often realized through a combination of thermal and mechanical stresses at high concentrations without any need for the use of chemicals. This also eliminates the need for additional separation or drying processes, which reduces the energy needed compared to conventional reactors [20]. The thermal and mechanical stresses applied to raw materials can be widely varied; hence, the molecular structures and functional properties of dietary fiber can be modified, as shown for orange [15,21], citrus [22], and sugar beet pulp [23], as well as onion waste [24].

Plant cell walls from by-products are the major contributors to the dietary fiber content. Depending on several factors such as plant taxonomy, plant organ, plant maturity, etc., the cell wall structure and composition and the structural details of cell wall polysaccharides (and non-carbohydrate polymers such as lignin) vary widely. However, the polymer/polysaccharide compositions and chemical structures are crucial for both the nutritional and functional properties of dietary fiber and by-products. Thermo-mechanical treatment affects the molecular weight and structure of dietary fiber polysaccharides; however, it is not well understood which polysaccharides and which structural elements are the most susceptible to stresses. Being dicotyledonous plants, apples have (primary) cell walls that are dominated by cellulose and pectin, with xyloglucans being the major but quantitatively less important hemicellulose [25].

Apple pomace is currently underutilized as animal feed or as feedstock for the production of bioenergy at producers’ expense, and only a small fraction from juice processing is valorized through pectin production [8,26,27]. Rarely, it is extruded as a minor ingredient in starch-based matrices to increase the fiber and bioactive content of the extruded products, such as breakfast cereals and snacks [28,29,30,31]. The functionalization of mere apple pomace by extrusion processing has been the topic of very few studies [32,33,34]. It has been shown that the yield of water-soluble polysaccharides can be increased by extrusion processing [32,34]. The shift from insoluble to soluble dietary fiber was explained by the solubilization of mostly non-pectinaceous cell wall polymers; also, partial degradation of the pectinaceous arabinogalactan side-chain was suggested [32]. Furthermore, Hwang et al. showed that hot water-based extraction of pectin from apple pomace can be facilitated by extrusion processing [33].

To the best of our knowledge, no study has been published to date on the changes of both the structure and the functional properties of apple pomace after extrusion processing. Therefore, this study focuses on the application of extrusion processing in order to modify the functional properties of apple pomace with special attention to the analysis of the impact of the process parameters and extrusion conditions on the morphological and structural characteristics of apple pomace.

## 2. Materials and Methods

### 2.1. Materials, Chemicals, and Reagents

Apple pomace (not enzymatically treated, from juice production) with a moisture content of 2.5 ± 0.3% (automatic Karl Fischertitration) was kindly supplied by Herbafood Ingredients GmbH (Company of H&F-Group, Werder (Havel), Germany). According to the supplier, the apple pomace was composed of 60% dietary fiber, 25% (digestible) carbohydrates, and 5% protein. Rapeseed oil was provided by Bernhard Schell GmbH (Lichtenau, Germany). Unless stated otherwise, all chemicals came from Carl Roth (Karlsruhe, Germany), Merck (Darmstadt, Germany), Sigma Aldrich (St. Louis, MO, United States) or VWR (Radnor, PA, USA). The heat-stable α-amylase Termamyl 120 L (EC 3.2.1.1, from *Bacillus licheniformis*, 120 KNU·g^−1^), the amyloglucosidase AMG 300 L (EC3.2.1.3, from *Aspergillus niger*, 300 AGU·g^−1^) and the protease Alcalase 2.5 L (EC 3.4.21.62, from *Bacillus licheniformis*, 2.5 AU·g^−1^) were supplied by Novozymes (Bagsværd, Denmark) and were used for the preparative isolation of dietary fiber. Heat-stable α-amylase (EC 3.2.11, from *Bacillus licheniformis*, 3000 U·mL^−1^), amyloglucosidase (EC 3.2.1.3 from *Aspergillus niger*) and protease (EC 3.4.21.14 from *Bacillus licheniformis*) (with all three enzymes being used for analytical dietary fiber analysis and the first two being used to determine starch contents), as well as endo-galactanase (EC 3.2.1.89 from *Aspergillus niger*) and endo-arabinanase (EC 3.2.1.99 from *Aspergillus niger*) were obtained from Megazymes (Bray, Ireland).

### 2.2. Extrusion Trials

A co-rotating, twin-screw extruder (Coperion ZSK 26Mc, Stuttgart, Germany) with a length-to-diameter ratio (L/D) of 29 and a screw diameter of 25.5 mm was used to perform the extrusion trials. The screw configuration contained kneading blocks and reverse transport elements (see [35] config.B). The extruder was equipped with a gravimetrically controlled feeder (DDW-DDSR40 from Brabender, Duisburg, Germany) and a piston-membrane pump (model KM 251, Alldos, Pfinztal, Germany). Material and water were fed to the first of the seven sections of the barrel, which could be heated independently. The last three sections were heated to 100 and 120 °C. A circular die of 3 mm diameter and 10 mm length was used. All trials were run at a constant total feed rate of 10 kg·h^−1^, whereas the water feed was varied among 4 kg·h^−1^, 2 kg·h^−1^ and 1.5 kg·h^−1^. The screw speed *n* was varied among 200, 450, and 700 min^−1^.

The processing conditions were characterized by the material temperature at the die exit (*T_M_*), and the specific mechanical energy (*SME*). *SME* (Wh·kg^−1^) was calculated according to Equation (1):

(1) $$SME\;=\;\frac{\frac{n}{n_{max}}\times \frac{M_{d}-\;M_{d,\;unload}}{100}}{\dot{m}}\;\times P_{max}$$

where n and $n_{max}$ are the actual and maximum screw speeds (1800 min^−1^), $M_{d}$ and $M_{d,\;unload}$ are the actual and idle torques (%), $\dot{m}$ represents the total mass flow (kg·h^−1^) and $P_{max}$ the maximum engine power (40 kW).

Samples were taken upon achieving steady-state conditions and then dried at 4 °C for 15 min. Extrusion trials were performed in duplicate.

Raw and extruded apple pomace was ground in a coffee mill (M55, Petra Electric, Ense, Germany) and sieved to a particle size of 0.14 mm < *x* < 0.28 mm. Afterwards, the samples were dried in a vacuum dryer (Heraeus, Hanau, Germany) at 25 °C and 8 mbar to constant mass. Most experiments to analyze the techno-functional properties of the raw material and extruded samples were performed using both the non-sieved samples and the fraction with a particle size of 0.14 mm < *x* < 0.28 mm, whereas the chemical analyses were only performed using the non-sieved samples.

### 2.3. Water Solubility Index and Water Absorption Index

The water solubility index (WSI) was determined by a modified method according to Anderson [36]. In a 50 mL centrifuge tube, 0.5 g of the sample ($m_{powder}$) was dissolved in 19.5 g demineralized water, mixed for 1 min with a vortex mixer and afterwards shaken for 24 h at 200 min^−1^ in a rotary shaker (Orbital shaker Incubator SI 50, Stuart, Staffordshire, United Kingdom) at room temperature. Samples were centrifuged at 4600× *g* for 50 min at 20 °C. Subsequently, the supernatant was removed and carefully placed into another tube. The precipitate and supernatant were dried in a drying oven at 80 °C for 72 h and weighed. WSI were calculated using Equation (2). On the assumption that the rigid cell wall would be loosened by extrusion processing, centrifugation would squeeze out loosely bound water. Thus, the water absorption index (WAI) was determined similarly to the common method (Anderson [36]) described above, but without the centrifugation step. Samples rested for 3 h to separate by sedimentation. WAI was calculated according to Equation (3).

(2) $$WSI\;=\;\frac{m_{supernatant,\;\;dried}}{m_{powder}}$$

(3) $$WAI\;=\;\frac{m_{preciptate,\;wet}-m_{preciptated,\;dried}}{m_{preciptated,\;dried}}$$

WSI and WAI of selected samples were measured for extrusion trials 1 and 2. No significant difference were observed. Therefore, the analyses of WSI and WAI were performed only in triplicate using one extrudate.

### 2.4. Oil Binding Capacity

The oil binding capacity (OBC) was determined by a modified method introduced by Caprez et al. [37]. Sample (0.5 g; prepared according to 2.2) was added to 10 g of rapeseed oil, vortexed for 1 min and shaken for 24 h (200 min^−1^, Universal Shaker SM30C, Edmund Bühler, Bodelshausen, Germany). Samples were centrifuged at 4250× *g* for 50 min at 25 °C. Subsequently, the supernatant was carefully transferred to another tube and weighed. The oil binding capacity was defined as the amount of oil that was absorbed by the sample, and was calculated by Equation (4).

(4) $$OBC\;=\frac{m_{oil,\;added}-m_{oil,\;removed}}{m_{powder}}$$

### 2.5. Rheological Properties

To characterize the rheological properties, 1 g of sample prepared according to the description in Section 2.2 was added to 10 g of demineralized water. The sample was stirred for 10 min and 200 min^−1^ (magnetic mixer) and stored at room temperature for 50 min, sealed with parafilm. Samples were measured in an Anton Paar MC 301 rheometer (Graz, Austria) using a parallel plate with a diameter of 50 mm and a gap of 2 mm. The measurement routine started with 90 s of rest. Afterwards, the sample was subjected to oscillatory shear measurements with an amplitude of 0.1% and a frequency of 1 Hz at 25 °C, which is in the linear viscoelastic region of the sample. To determine the complex viscosity η*, a mean value of nine measurement points of each measurement was considered. The measurements were performed in triplicate using one extrudate.

### 2.6. Chemical Analysis

All data are presented as mean ± standard deviation (*n* = 3 using one extrudate). All data from structural analyses of isolated dietary fiber (molecular weight distribution, monosaccharide composition and linkage, arabinan and galactan oligosaccharides) are given as mean ± range/2 (*n* = 2).

**Free mono- and disaccharides.** Water (10 mL) was added to 200 mg of sample in a 15 mL tube. The suspension was vortexed and treated for 10 min in an ultrasonic bath (temperature < 30 °C). After 5 min, the solution was centrifuged for 10 min, and the supernatant was collected in a 50 mL volumetric flask. The extraction steps were repeated four times. The combined extracts were adjusted to 50 mL, and a 2 mL aliquot was filtered through a PTFE membrane filter (45 µm). A 200 µL aliquot was mixed with 800 µL of ethanol; precipitation was completed within 10 min. After centrifugation, the supernatant was transferred and evaporated. The residue was redissolved in 200 µL of water, 20 µL of d-fucose (200 µM) was added as internal standard, and the solution was analyzed using HPAEC-PAD [38].

**Starch content.** The starch content was determined after enzymatic digestion by analysis of liberated glucose using HPAEC-PAD. The sample (1 g) was suspended in 50 mL of 0.08 M phosphate buffer (pH 6.0), and the suspension was heated for 15 min at 60 °C. Thermostable α-amylase (1 mL) was added, and the suspension was heated for 15 min at 92 °C. After cooling to room temperature, the pH was adjusted to 4.5, 100 µL of amyloglucosidase solution (10 mg·100 µL^−1^) was added, and the suspension was heated for 15 min at 60 °C, followed by adjusting the volume to 100 mL. To an aliquot of 4 mL, 1 mL of Carrez I (150 g·L^−1^ K_4_[Fe(CN)_6_]·3H_2_O) and 1 mL of Carrez II (300 g·L^−1^ ZnSO_4_·7H_2_O) solutions were added, and the pH was adjusted to 7.5–8.0. Following centrifugation and volume adjustment, the solution was diluted for analysis by HPAEC-PAD and 20 µL of the internal standard d-Fucose (200 µM) was added. Monosaccharide (glucose) analysis was performed as previously described by Wefers, Gmeiner et al. [38]. The starch content was calculated as the sum of anhydroglucose.

**Dietary fiber analysis.** To determine the insoluble, soluble and low-molecular-weight soluble dietary fiber contents excluding resistant starch, a combination of the procedures AOAC 985.29 [39] and AOAC 2009.01 [40] was performed. In brief, the ground material (1 g; particle size < 0.5 mm) was first digested with a thermostable amylase (pH 6.0, 20 min, 92 °C), then with a protease (pH 7.5, 30 min, 60 °C) and finally with an amyloglucosidase (pH 4.5, 30 min, 60 °C). The warm solution was filtered through Celite crucibles. Residues were washed with water, ethanol and acetone. The residue remaining in the crucible corresponded to the uncorrected insoluble dietary fiber and could be weighed after drying overnight at 105 °C. The filtrate and water washing were combined, and soluble dietary fiber was precipitated by adding four times the volume of ethanol. The residue was separated by filtration through crucibles and washed with ethanol and acetone. This residue, after drying at 105 °C, corresponded to the uncorrected soluble dietary fiber. Both insoluble and soluble dietary fiber contents were corrected for ash (gravimetrically after combustion at 520 °C) and residual protein contents (ammonia detection using an ammonia electrode after Kjeldahl digestion; N × 6.25 [41]). The filtrate obtained from the isolation of soluble dietary fiber was evaporated to almost dryness by rotary evaporation, redissolved in water, adjusted to a defined volume and desalted on a column filled with Amberlite FPA53/Ambersep 200 (Megazyme, Bray, Ireland). Elution was carried out with water. The eluate was evaporated and redissolved in a defined volume of water. Low-molecular-weight dietary fiber oligosaccharides were separated from mono- and disaccharides and quantitated by HPLC (Hitachi, Merck, Darmstadt, Germany) with refractive index (RI) detection (Knauer, Berlin, Germany) using a TSKgel G2500PWxl column (300 mm × 7.8 mm, 7 μm particle size, Tosoh, Tokyo, Japan). The elution was performed with water at 60 °C using a flow rate of 0.4 mL·min^−1^. The boundary (elution time) between di- and oligosaccharides was determined by analysis of maltose and maltotriose. Quantification was performed using glycerol, which was added before the first filtration step as an internal standard; d-glucose was used to determine the response factor.

**Preparative dietary fiber isolation.** The isolation of soluble and insoluble dietary fiber was carried out using the principles of the AOAC 985.29 method (enzymatic treatment using thermostable α-amylase, protease, and amyloglucosidase); however, it was modified to allow for a separation of soluble and insoluble dietary fiber on a preparative scale, as described by Bunzel et al. [42]. The structural analyses described below were performed using these dietary fiber preparations.

**Molecular weight distribution.** To determine the molecular weight distribution of soluble fiber polysaccharides, soluble dietary fiber samples were dissolved in 50 mM sodium nitrate solution (2 mg·mL^−1^) within 24 h at 40 °C. These were analyzed by HPLC-RI (Hiatchi, Merck, Darmstadt, Germany) using a TSK-gel G4000PWxl column (300 mm × 7.8 mm, particle size 10 µM, Tosoh). Sodium nitrate (50 mM) was used as eluent, and a flow rate of 0.5 mL·min^−1^ and a temperature of 40 °C were maintained. Calibration was performed using dextrans as standard polysaccharides.

**Polysaccharide composition.** The monosaccharide composition of insoluble, soluble (and low-molecular-weight soluble; obtained from the analytical dietary fiber approach) dietary fiber carbohydrates was determined as described by Wefers, Gmeiner et al. [38]. In brief, insoluble dietary fiber polysaccharides were hydrolyzed by using sulfuric acid hydrolysis, whereas soluble dietary fiber polysaccharides were hydrolyzed by using methanolysis followed by trifluoroacetic acid (TFA) hydrolysis. Liberated monosaccharides were analyzed by HPAEC-PAD. This procedure was modified to analyze the monosaccharide composition of the low-molecular-weight soluble dietary fiber carbohydrates. These were preparatively purified by HPLC (Hiatchi, Merck, Darmstadt, Germany) with RI detection (Knauer, Berlin, Germany) using a TSKgel G2500PWxl column. The elution was performed with water at 60 °C and a flow rate of 0.4 mL·min^−1^. The elution window (16 to 38.6 min) of the oligosaccharide fraction was adopted from the analysis of low-molecular-weight (ethanol) soluble dietary fiber described above. Collected solutions were evaporated and re-dissolved in a defined volume of water. An aliquot of this solution (20 µL) was evaporated and treated with 500 µL of a 2 M TFA solution for 1 h at 121 °C, followed by evaporation. Twice, ethanol was added and evaporated. The residue was dissolved in 180 µL of water, 20 µL of 1 mM d-mannitol was added and the solution was analyzed using HPAEC-PAD.

**Polysaccharide linkage analysis.** The analysis of interunit linkages of insoluble and soluble fiber polysaccharides was carried out as described by Gniechwitz et al. [43]. In brief: methylation using methyl iodide in DMSO/NaOH; extraction into dichloromethane; hydrolysis using 2 M TFA at 121 °C for 90 min; reduction using NaBD_4_; acetylation in acetic anhydride using 1-methylimidazole as catalyst; identification of partially methylated alditol acetates by GC-MS; and semiquantitation by GC-FID using a molar response factor as described by Sweet et al. [44].

**Arabinan and galactan screening.** Arabinans and galactans as neutral pectic (rhamnogalacturonan I) side chains were analyzed by using a profiling approach established by Wefers, Bunzel et al. [45]. In brief, after enzymatic digestion with *endo*-arabinanase and *endo*-galactanase, the arabinan and galactan oligosaccharides were determined semiquantitatively using HPAEC-PAD.

**Degree of (pectin) esterification.** To measure the degree of esterification, polymer-bound galacturonic acid was analyzed photometrically using the method described by Blumenkrantz et al. [46]. The methanol content was measured by NMR after alkaline hydrolysis. Sodium hydroxide solution in D_2_O (2 M, 1 mL) and 0.1 mL of a 0.2 mg·mL^−1^ 3-(trimethylsilyl)propionic-2,2,3,3-*d*_4_ acid sodium salt solution in D_2_O (as an internal standard) were added to 15 mg of sample material. The hydrolysis was carried out for 2 h in an ultrasonic bath. After centrifugation and membrane filtration (PTFE; 0.45 µm), the solution was analyzed using ^1^H-NMR spectroscopy (Avance 500 MHz instrument with a Prodigy cryoprobe; Bruker, Rheinstetten, Germany). A standard Bruker ^1^H-NMR pulse program (zg30) was used, and the following parameters were applied: 65,536 data points, acquisition time 3.28 s and relaxation delay (D1) 35 s. Processing was done using zero filling, a by factor of two and application of a Lorentz function (EM, 0.3 Hz).

### 2.7. Simons’ Stain

Simons’ stain method was performed according to the *Modified Simons’ stain technique* described by Chandra et al. [47]. Instead of Direct Blue 1 and Direct Orange 15, Direct Red 28 (DR 28, Congo Red, CAS: 573-58-0, Carl Roth GmbH & Co. KG, Karlsruhe, Germany) and Direct Yellow 4 (DY 4, CAS: 3051-11-4, Sigma-Aldrich Chemie GmbH/Merck KGaA, Munich, Germany) were used [48,49]; because in their study, DR 28 and DY 4 provided the most accessible surface values. Dye concentration was 1% (*w*/*w*). The measurements were performed in triplicate using one extrudate.

## 3. Results and Discussion

### 3.1. Influence of Extrusion Parameters on the Extent of Thermo-Mechanical Treatment

**Figure 1:**
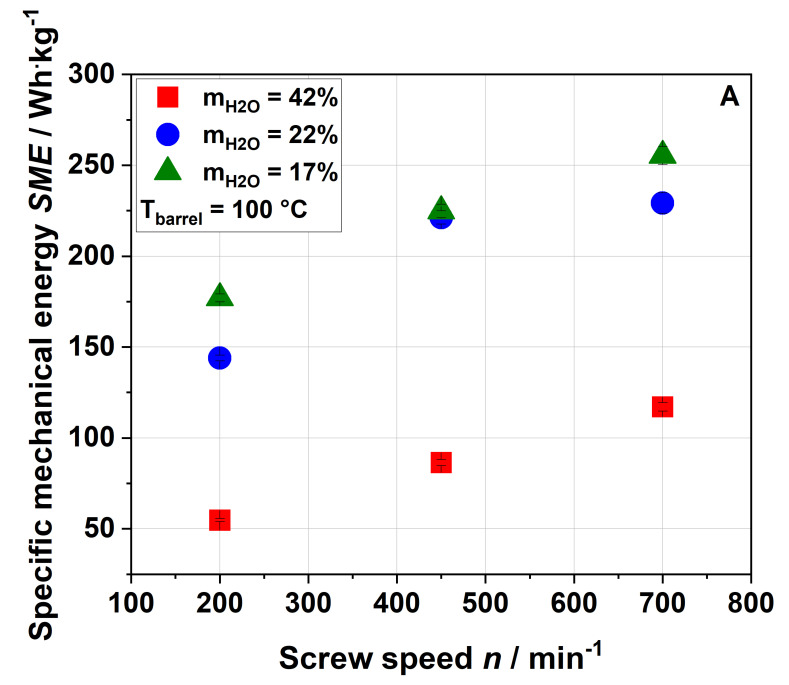

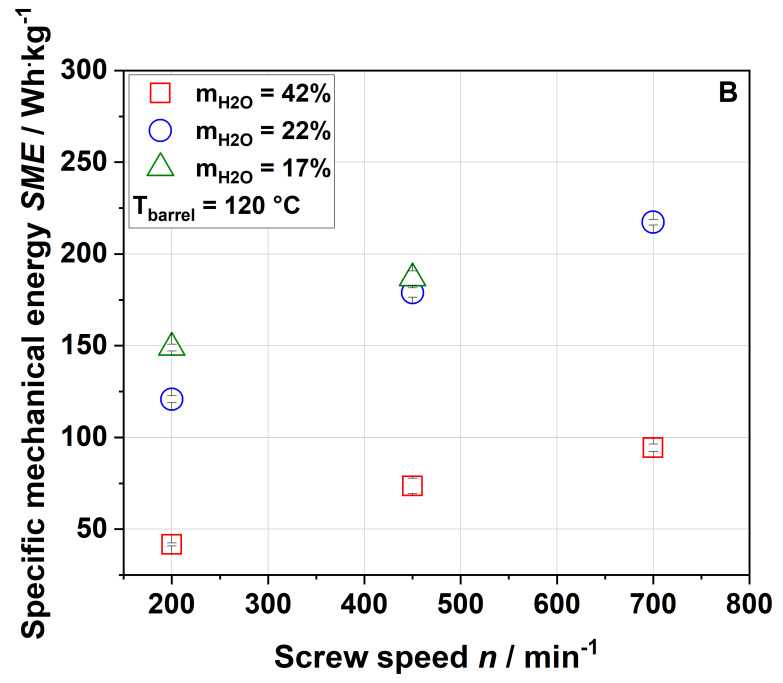
Effect of screw speed and water content on the specific mechanical energy (SME) at (**A**) 100 °C barrel temperature and (**B**) 120 °C barrel temperature.

**Figure 2:**
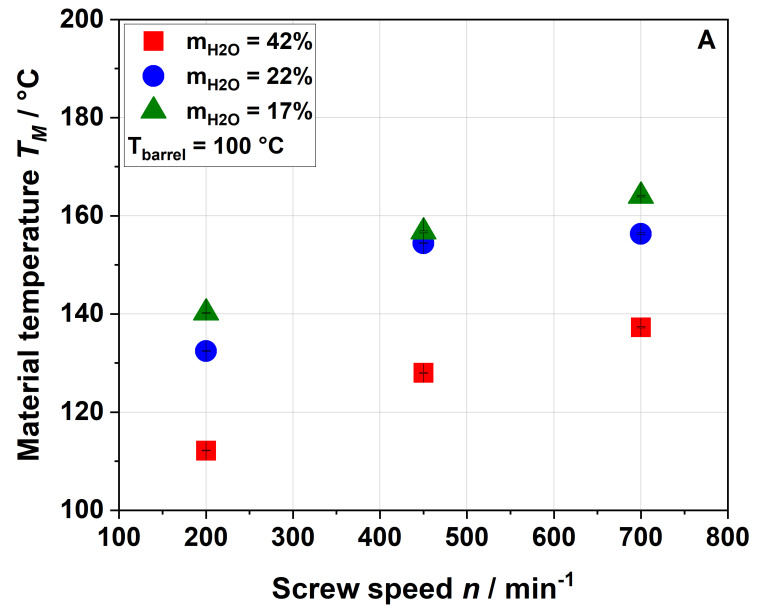

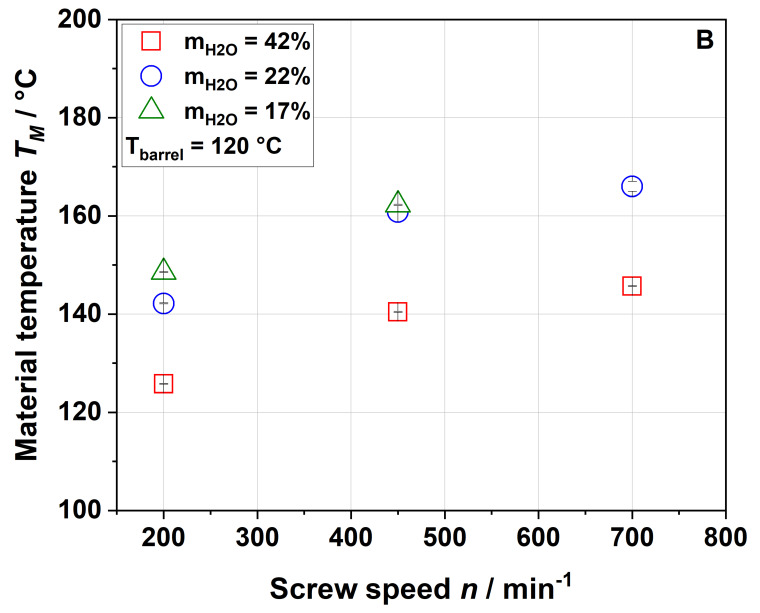
Effect of screw speed and water content on the material temperature (*T*_M_) measured at the die entrance at (**A**) 100 °C barrel temperature and (**B**) 120 °C barrel temperature.

To better understand and control the impact of extrusion parameters (e.g., screw speed, barrel temperature and water content) on the modification of the functional properties of apple pomace, their influence on the resulting processing conditions, namely the thermal and mechanical stress profiles, were analyzed. For this purpose, the specific mechanical energy (SME) and material temperature (T_M_) were used as indicators (Figure 1 and Figure 2).

As expected, an increase in screw speed led to an increase in SME. Reducing the water content from 42% to 22% resulted in a strong increase in SME due to the higher viscosity of the melt, whereas a further decrease in water content, i.e., to 17%, led to a slight increase only or even to no further increase in SME. A barrel temperature of 120 °C (Figure 1B) resulted in a lower SME at the same screw speed and water content compared to a barrel temperature of 100 °C (Figure 1A). This was potentially due to the temperature dependence of the matrix viscosity (lower viscosity at higher temperature) resulting in lower mechanical shear stresses [50]. No samples were taken at a water content of 17%, barrel temperature of 120 °C and screw speed of 700 min^−1^ due to the instability of the process.

Figure 2 shows that the material temperature was always higher than the barrel temperature, indicating that viscous dissipation (resulting from the mechanical energy) plays an important role in the extrusion process of apple pomace.

The rising screw speed further increased the material temperature. Also, decreasing the water content from 42% to 22% resulted in a significantly higher material temperature, whereas a further decrease to 17% had only a minor impact (cf. the SME data shown in Figure 1). Again, this suggests that heating of the material was mainly driven by the viscous dissipation coupled to mechanical stress. As expected, an increase in the barrel temperature from 100 °C (Figure 2A) to 120 °C (Figure 2B) resulted in a higher material temperature.

### 3.2. Influence of Extrusion Conditions on Functional Properties

Fibers can impart favorable functional properties upon foods. Most food matrices are water-based, and therefore, the functional properties regarding the interaction of fibers with water are crucial. The water solubility index describes the amount of fiber which solubilizes in water. The amount of water that can be absorbed by a sample is defined as the water absorption index. These indices are essential to adjust textural properties like viscosity in yogurts, to avoid syneresis in dairy products or to maintain the moisture in, e.g., bread or sausages. Water absorption also plays a major role in the digestion of foods. Some foods are characterized by a high amount of fat-like creams or emulsions. Therefore, also the oil-binding capacity was determined.

The results in Section 3.2.1, Section 3.2.2, Section 3.2.3, Section 3.2.4 describe the data obtained from using the sieved fraction of the raw material and the extruded samples (particle size of 0.14 mm< *x* < 0.28 mm).

#### 3.2.1. Water Solubility

**Figure 3:**
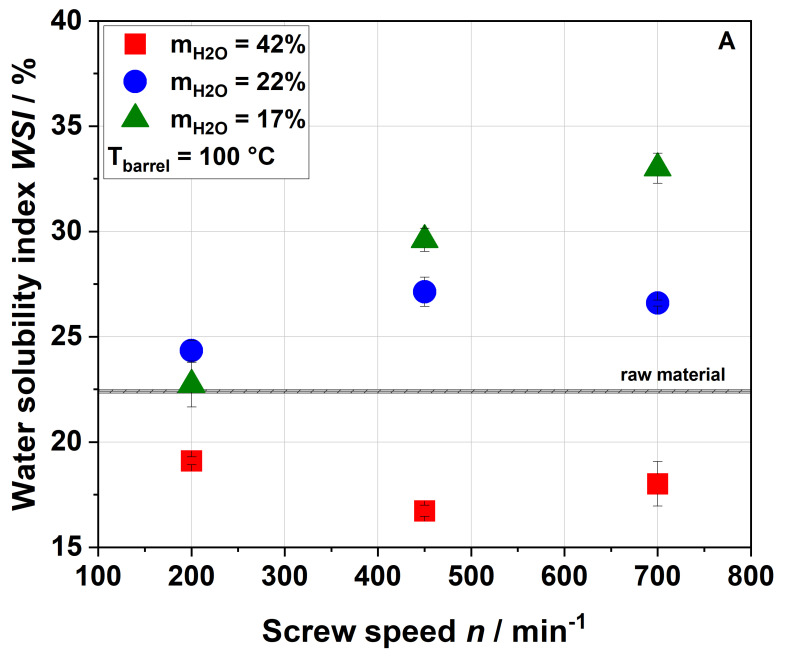

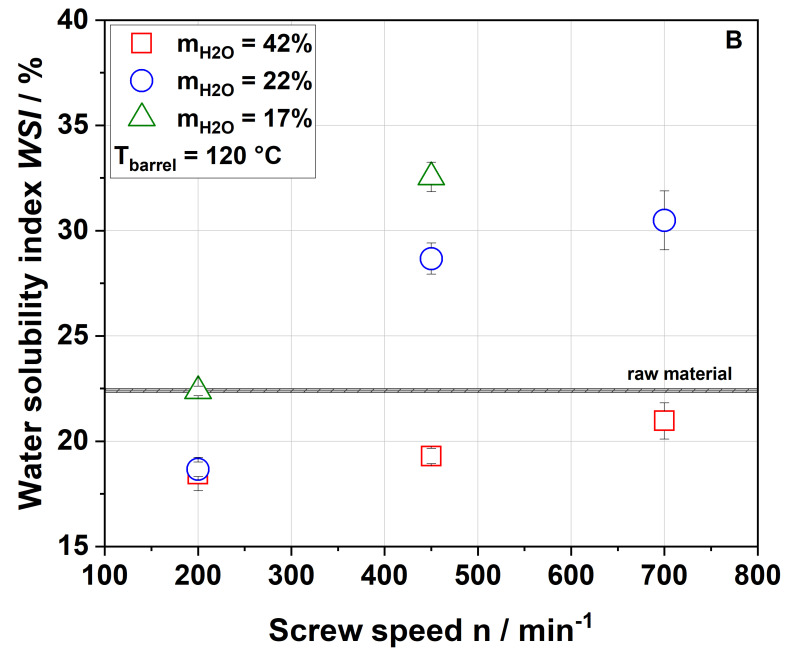
Effect of screw speed and water content on the water solubility index (WSI) at (**A**) 100 °C barrel temperature and (**B**) 120 °C barrel temperature. Values of the raw material are marked as a black line, whereas values of extruded samples are given as dots.

Figure 3 shows the influence of screw speed on the water solubility index (WSI) of raw and extruded apple pomace.

For a water content of 42%, the WSI of the extruded samples was lower than that of the raw material for all screw speeds and barrel temperatures investigated. This can be explained either by agglomeration due to extrusion processing or by structural changes (see Section 3.3). For water contents of 17% and 22% and a barrel temperature of 100 °C, the WSI of raw and extruded samples were roughly comparable at 200 min^−1^, whereas an increase in screw speed resulted in a higher water solubility of the extruded samples. The highest WSI was achieved at the lowest water content and highest screw speed, at which both SME and T_M_ reached their maxima (see Figure 1).

A similar trend was also observed by Hwang et al. [32] and Liu et al. [34], although they reported significantly higher values of WSI for both raw material (22.4–35%) and extruded apple pomace (49.6–57.8%). These deviations may have been due to differences in the raw material (e.g., enzymatically vs. not enzymatically treated pomace) and the processing parameters (e.g., different extruder and screw configurations) used. The above authors related the increase in WSI to the solubilization of the rigid cell wall structure, which was caused by increasing mechanical stresses [32]. However, a parallel increase in material temperature was not discussed. Chang et al. showed that thermal treatment only (achieved by autoclaving) also resulted in higher amounts of soluble fibers [51], which indicated that thermal stresses could be equally important for an increased solubility of apple pomace at higher screw speeds.

The WSI data of the samples extruded at a higher barrel temperature (Figure 3B) demonstrate higher water solubilities at screw speeds of 450 and 700 min^−1^. This was potentially related to the higher thermal treatment. Analysis of the processing conditions (Figure 1 and Figure 2) showed that increasing the barrel temperature resulted in higher material temperatures but lower SME, again suggesting that thermal stresses are important for the modification of apple pomace.

Apple pomace is mainly composed of cellulose, hemicelluloses and pectins, with cellulose being insoluble. Unlike cellulose, hemicelluloses and pectins are partially water soluble. An increase in water solubility was often attributed to increasing amounts of water-soluble pectic and hemicellulosic polymers [32], which was also shown for lemon fibers [52,53]. Previous studies also showed that extrusion may result in lower portions of arabinose and galactose in the water-insoluble residues indicating a solubilization of pectic substances during extrusion processing [23,32].

#### 3.2.2. Water Absorption

**Figure 4:**
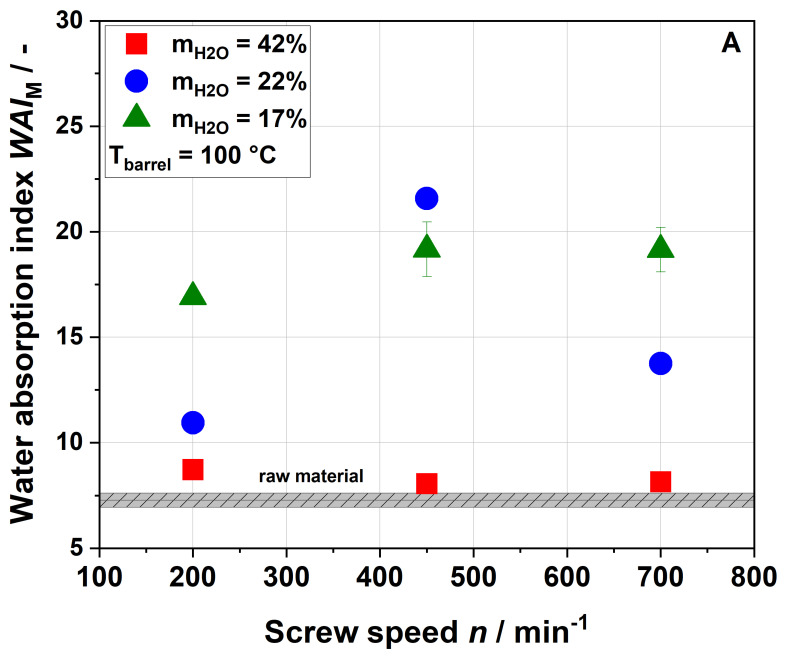

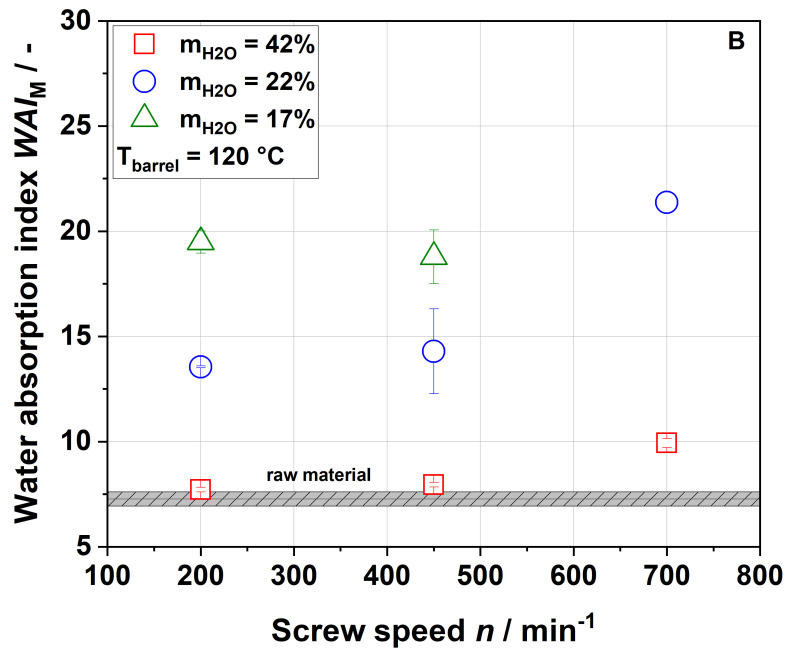
Effect of screw speed and water content on the modified water absorption index (WAI) at (**A**) 100 °C barrel temperature and (**B**) 120 °C barrel temperature.

Samples extruded at water contents of 17% and 22% showed a higher water absorption index (WAI) than the raw material, whereas samples that were processed at the highest water content of 42% showed no significant change in WAI regardless of the screw speed applied (Figure 4A,B).

At a water content of 17% and a barrel temperature of 100 °C, an increase in screw speed from 200 to 450 min^−1^ led to higher WAI. At a water content of 22%, the maximum WAI was observed for a screw speed of 450 min^−1^. At a higher barrel temperature of 120 °C, WAI differed depending on the water contents during extrusion (17% or 22%) at all screw speeds, with higher WAI at a water content of 22%.

In the literature, for other by-products, mostly decreases in WAI with increasing thermo-mechanical treatment have been reported [24,54]. This can be explained by the centrifugation step used in these studies. In order to identify the exact amount of water that can be taken up by a sample, no centrifugation step was performed in this study. The increase in WAI was most likely due to changes in the cell wall structure, which might be opened up during extrusion processing. Higher porosity results in absorbing more water during rehydration [55], as discussed in more detail in Section 3.4.

#### 3.2.3. Oil-Binding Capacity

**Figure 5:**
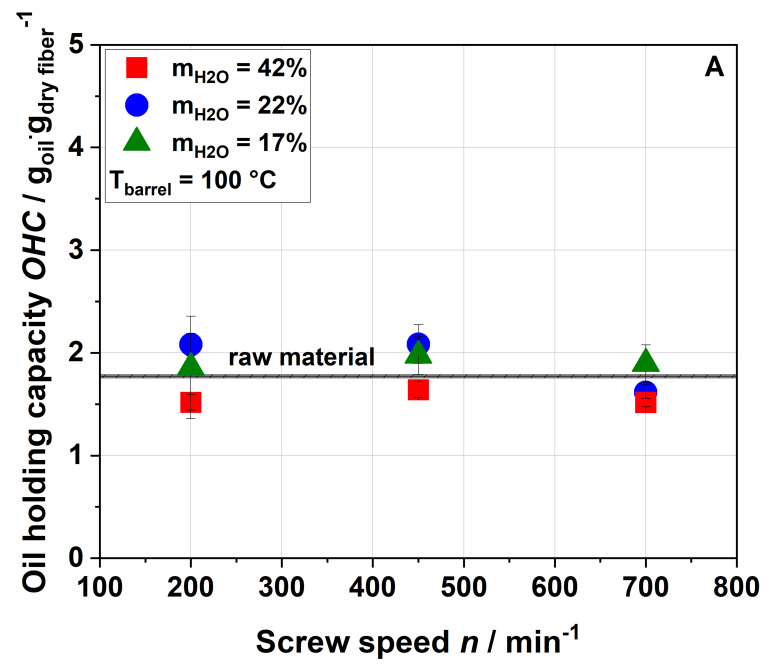

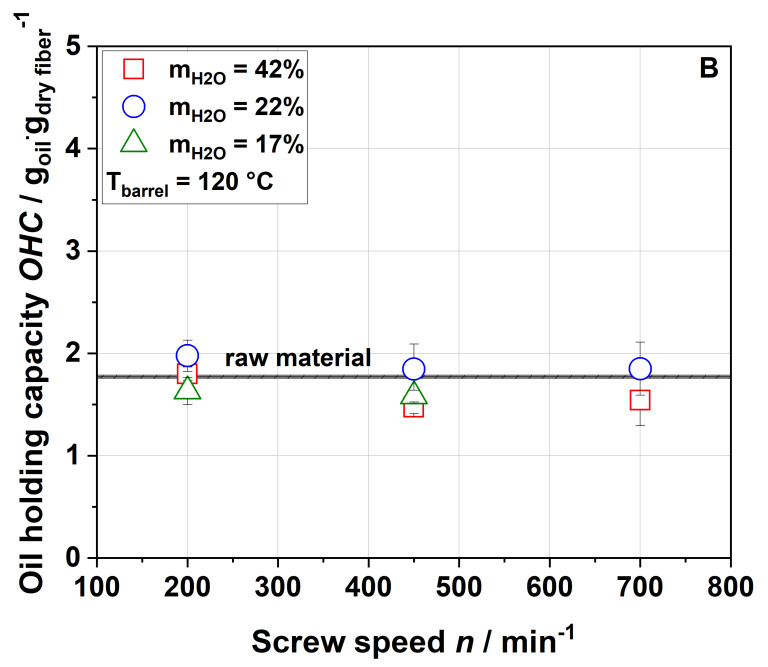
Effect of screw speed and water content on the oil-binding capacity (OBC) at (**A**) 100 °C barrel temperature and (**B**) 120 °C barrel temperature.

The oil-binding capacity (OBC) of raw and extruded apple pomace is displayed in Figure 5 as a function of the screw speed, water content and barrel temperature.

Figure 5 shows that extrusion treatment did not change the OBC of the samples. A low OBC value (1.23), which did not significantly change after extrusion treatment, is also reported for orange pomace [21]. Although these results suggest that extrusion processing did not result in major changes of the lipophilic functional groups of cell wall molecules, the degree of pectin methylation and acetylation decreased with increased thermo-mechanical stress, as detailed in Section 3.3.

#### 3.2.4. Gelation Properties

**Figure 6:**
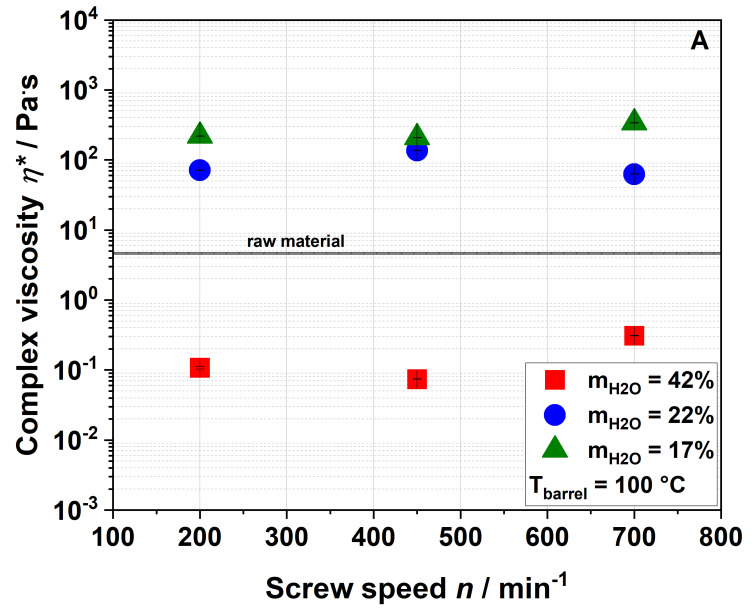

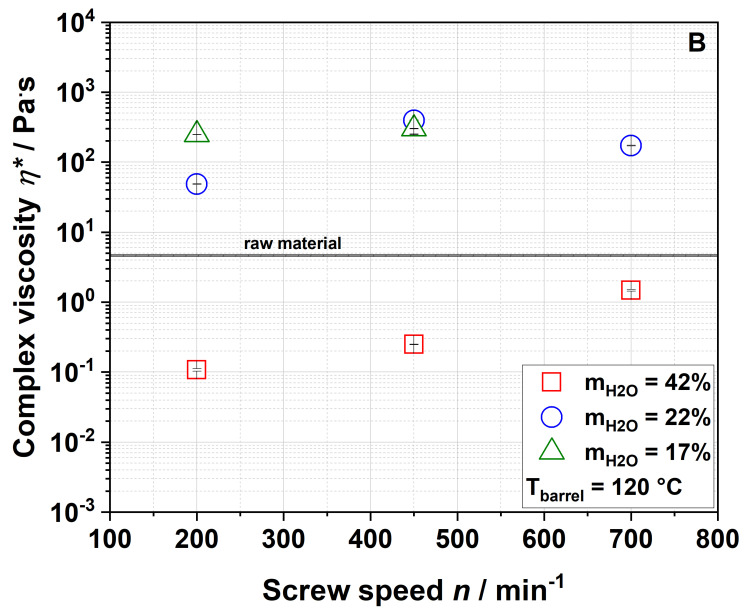
Effect of screw speed and water content on complex viscosity η* at (**A**) 100 °C barrel temperature and (**B**) 120 °C barrel temperature.

The effect of extrusion processing on the paste viscosity of water dispersions of apple pomace is depicted in Figure 6.

**Figure 7:**
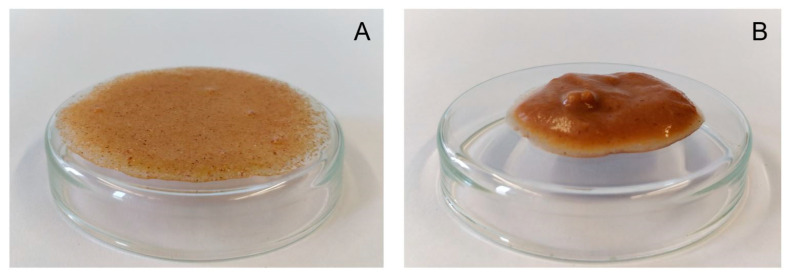
Water dispersions of 10% apple pomace powder extruded at 42% water content (**A**) and 17% water content (**B**). Other extrusion processing conditions were kept constant (200 min^−1^, barrel temperature 100 °C).

A water dispersion of the raw material had a complex viscosity of 4.6 Pa·s (Figure 6). Samples extruded at a water content of 42% formed even weaker pastes than raw apple pomace which, however, increased with screw speed at an elevated barrel temperature of 120 °C (Figure 6B). Samples that were extruded at lower water contents showed higher complex viscosities than the raw material. By decreasing the water content to 22% during extrusion, the complex viscosity of the samples extruded at a screw speed of 200 min^−1^ (barrel temperature: 100 °C) increased to 71.1 Pa·s. Under these conditions, a further reduction of the water content to 17% led to a complex viscosity of 218.6 Pa·s. Figure 7 shows pictures of the water dispersions of apple pomace extruded at water contents of either 42% or 17%, demonstrating different gelling behaviors.

Apparently, complex viscosity is not linearly dependent on the screw speed. Nevertheless, the results show that complex viscosity can significantly be increased (up to 50 times) by extrusion processing.

According to Ralet et al., paste formation is caused by solubilized pectic polymers [53]. Our results also demonstrate a correlation between paste viscosity and WSI. However, the WAI needs to be considered, too. Samples that take up more water swell more extensively, thereby increasing their specific volume. This leads to a larger excluded volume, which is not accessible by other particles. In consequence, there is less free volume (i.e., the dispersed volume fraction increases), resulting in an increased viscosity [56].

The paste-forming behavior of the samples can therefore be explained by increased water solubility and/or water absorption behavior. Extrusion disrupts the rigid cell wall structure (as described below), leading to a higher water uptake and swelling of the particles. At the same time, there is a larger number of soluble molecules (results of WSI see Section 3.2.1) in the direct neighborhood of dispersed particles, contributing to network formation.

#### 3.2.5. Influence of Particle Size on Functional Properties

With respect to functional properties, it is important to compare fractions that contain particles of similar size in order to avoid the influence of particle size on, for example, water absorption [57]. Therefore, all data presented so far were analyzed for a specific particle fraction (0.14 mm < x < 0.28 mm). In contrast, it is assumed that industrial applications will utilize the entire sample and not a specific particle size fraction. Therefore, this study also analyzed the influence of extrusion processing on the structure and composition, as well as on the functional properties of the ground but non-sieved sample material. The particle size distribution was as follows: The percentage of particles with a particle size < 14 mm was 9–11%, particle sizes 14–28 mm was 66–82% and particle size > 28 mm was 9–26%. As sieving may result in the separation of specific sample components (starch, for example, may be enriched in fractions of smaller particle size), the influence of particle size on functional properties is presented below. However, based on the data presented so far, we analyzed only the samples with the most interesting functional properties (barrel temperature 100 °C; water content 22%, and screw speeds of 200, 450, and 700 min^−1^ as well as water content of 42% and screw speed of 200 min^−1^). Hereafter, these samples will be designated as 100-42-200; 100-22-200; 100-22-450; and 100-22-700 (barrel temperature-water content-screw speed).

**Figure 8:**
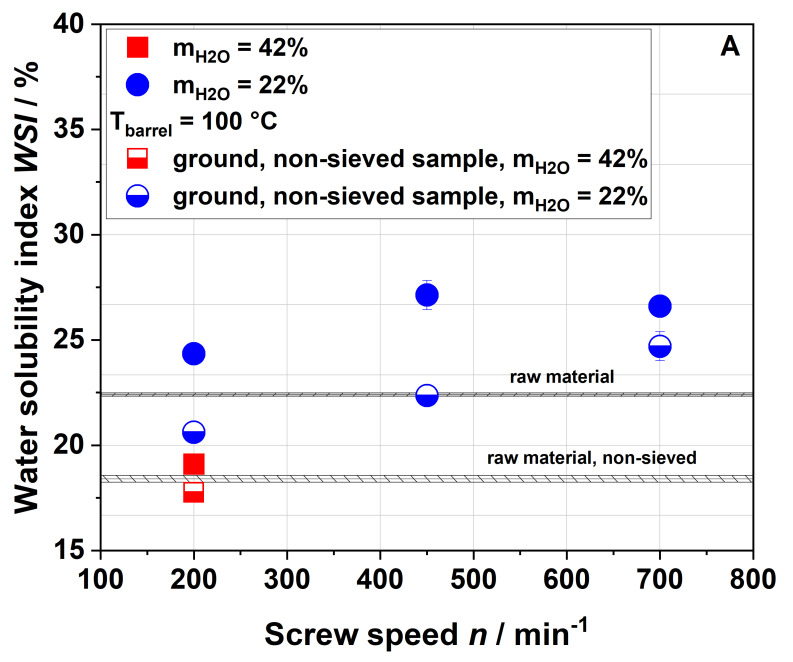

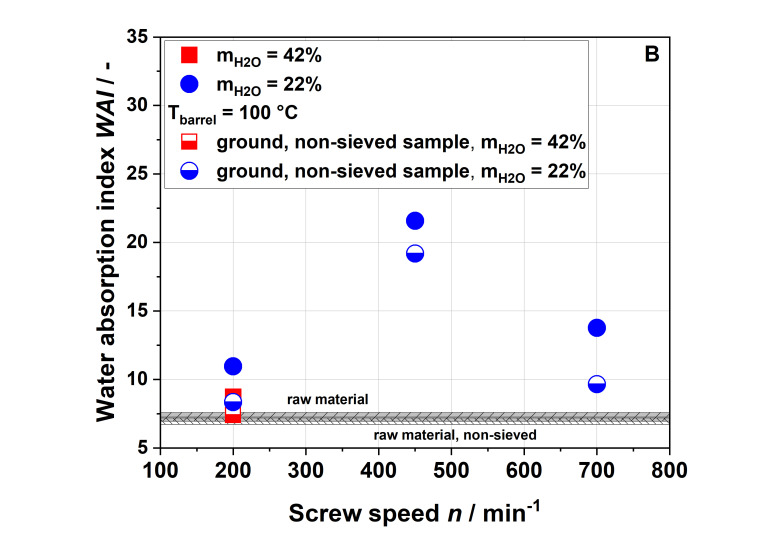

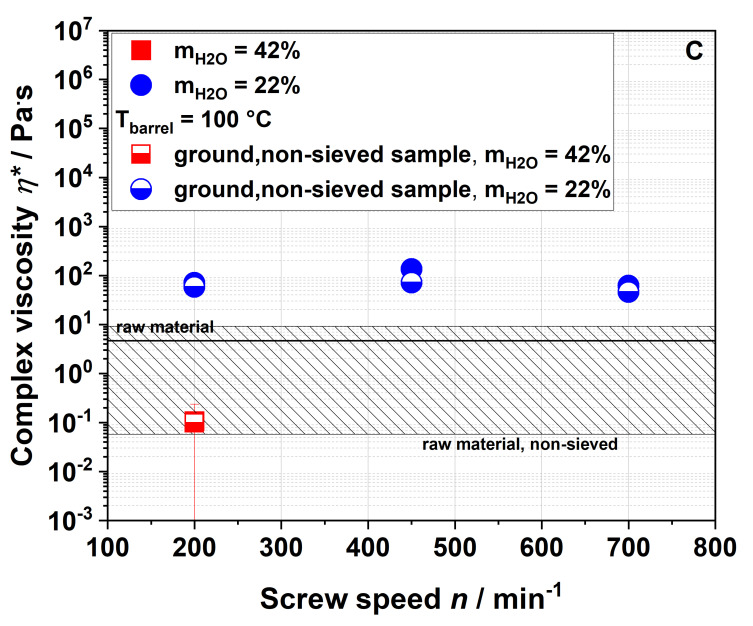
Water solubility index (**A**), water absorption index (**B**), and complex viscosity (**C**) of raw and extruded apple pomace for a particle fraction of 0.14–0.28 mm (filled) and the entire ground sample (half-filled).

Figure 8 shows the WSI (A), WAI (B) and complex viscosity (C) of raw and extruded apple pomace comparing the entire, ground, non-sieved material with the sieved fraction that has been described so far.

The WSI of all samples with a defined particle size of 0.14–0.28 mm was higher compared to those of the entire (non-sieved) samples. The WSI of the sample extruded with a water content of 42% and a screw speed of 200 min^−1^ was lower than the WSI of the non-sieved raw material. For a water content of 22%, the WSI increased with the screw speed also for the non-sieved samples. Although lower WSI values were observed for the entire sample as compared to the sieved fraction, the same trends were noted. The same holds true for the WAI (Figure 8B) and the complex viscosity (Figure 8C). However, when measuring the complex viscosity, non-sieved samples with low viscosity show a large standard deviation due to their broad particle size distribution.

### 3.3. Impact of Extrusion on Molecular Structures

The four samples 100-42-200, 100-22-200, 100-22-450 and 100-22-700 (see Section 3.2.5) were used to analyze the macroscopic and molecular structures of apple pomace before and after extrusion. To describe extrusion-based changes independently of the particle size and to characterize the application-relevant material, a detailed structural characterization was performed using the entire ground fraction as described in Section 3.2.5. However, to roughly check whether sieving has an impact on the polymer chemical composition, the monosaccharide composition of the polysaccharides of the sieved fraction (particle size of 0.14–0.28 mm) was also determined (after both sulfuric acid hydrolysis and methanolysis). The data (Supporting Information, Table A1) did not indicate major compositional differences between the sieved and the non-sieved samples, disapproving the possibility that, for example, starch had been systematically sieved out due to the formation of smaller particles during grinding.

**Protein, fat, ash content.** The methods and data of the analysis of protein, fat and ash are given in the Supporting Information (Table A2) simply to characterize the raw material and extruded products. Below, we will focus on carbohydrates.

**Free mono- and disaccharides.** Because low-molecular weight compounds may also contribute to the physicochemical properties of the pomace, mono- and disaccharides were analyzed. Glucose, fructose, sucrose and maltose were detected and quantified as free mono- and disaccharides (Supporting Information, Table A3). The glucose content (2.8 ± 0.3 g/100 g dry matter (DM)) did not change significantly during extrusion. In contrast, and as expected from the literature [58], the fructose content decreased with increasing thermo-mechanical treatment (from 8.8 ± 0.3 g/100 g DM to 3.4 ± 0.1 g/100 g DM (100-22-700)). Also, the sucrose content decreased from 2.3 ± 0.4 g/100 g DM to 0.7 ± 0.5 g/100 g DM (100-22-700), and maltose contents decreased independently of the extrusion conditions from 1.4 ± 0.3 g/100 g DM to below the limit of quantitation.

**Starch.** Depending on the stage of maturity, apples and thus apple pomace contain significant amounts of starch. The raw material contained 11.6 ± 0.7 g starch/100 g DM. Starch contents decreased to 8.5–9.2 g/100 g DM during extrusion (Table A4, Supporting Information) with the decline being widely independent of the extrusion conditions.

**Dietary fiber composition.** Insoluble dietary fiber contents decreased independently of the extrusion conditions (Table 1). Considering extrusion conditions using 22% water, soluble dietary fiber contents increased with increasing thermo-mechanical treatment. This trend was consistent with WSI data, although it needs to be considered that dietary fiber data exclude mono- and disaccharides, starch and protein, which are included in the WSI assay. Low-molecular-weight soluble dietary fiber was only formed at a screw speed of 700 min^−1^, which can easily be explained by extensive polymer degradation under high thermo-mechanical stress.

Unlike in the case of the WSI data and also in disagreement with the applied thermo-mechanical stress, the soluble dietary fiber content was higher for the 100-42-200 sample than for the 100-22-200 sample. A simple explanation is difficult to find; however, Maillard reaction products that are partially captured using the dietary fiber methodology might be at least one factor contributing to this unexpected finding.

The dietary fiber contents of apple pomace have been determined in the past [7,59]. According to the literature, insoluble and soluble fiber contents are dependent on the variety of apples (insoluble dietary fiber ranges between 33–67%, soluble fiber between 3–14%). The data analyzed here are within the range of the values found in the literature. Besides taking into account the variety, it needs to be considered that fiber contents and composition also depend on tissue maturity and changes during storage [25,60]. The influence of different extrusion parameters on fiber contents has been previously studied by Hwang and coworkers [32], who also found decreasing insoluble dietary fiber and increasing soluble fiber contents due to extrusion.

**Figure 9:**
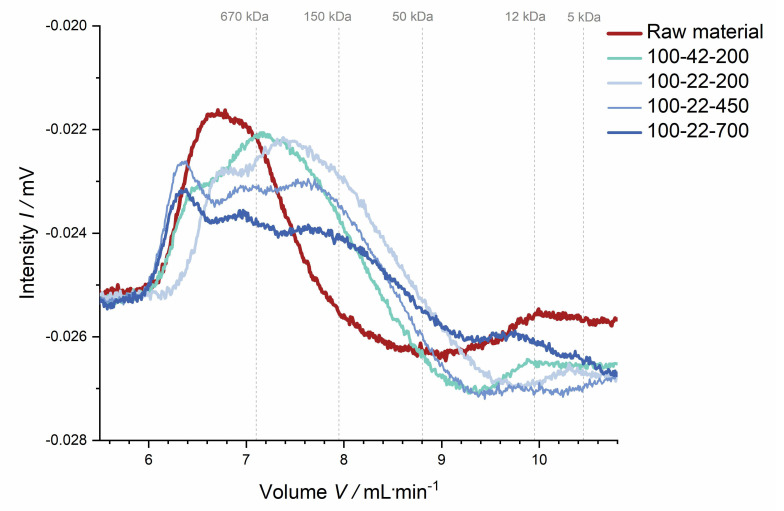
Molecular weight distribution of the soluble dietary fiber of raw and extruded apple pomace. The molecular weight distribution was determined by high-performance liquid chromatography with a refractive index detector. The intensity (refractive index detector signal) was plotted against the elution volume. Molecular weight calibration was performed by using dextrans (gray dotted lines).

**Polysaccharide characterization.** Dietary fiber data indicate that thermo-mechanical treatment influences the structure of fiber polysaccharides, potentially going along with polymer solubilization and more or less specific degradation. This hypothesis is supported by data from the molecular weight distribution analysis. This analysis was used to assess the extent of structural changes of the soluble dietary fiber fraction rather than determining the exact molecular weight. Figure 9 shows that the molecular weight distribution became broader with increasing thermo-mechanical treatment, suggesting that large polymers had been (partially) broken down, thus backing up the dietary fiber composition data described above.

The monosaccharide compositions of insoluble fiber polysaccharides of raw material and extruded samples were determined after sulfuric acid hydrolysis (Table 2). Glucose was the main monomer (43.4 mol%) in the raw material, followed by arabinose (17.3 mol%), galacturonic acid (11.9 mol%), xylose (11.0 mol%) and galactose (10.1 mol%). The main portion of glucose could be attributed to cellulose, as well as being part of the hemicellulose xyloglucan. Xyloglucans, xylogalacturonans and secondary cell wall xylans are potential sources of xylose. Mannans are quantitatively less important in apple pomace insoluble fiber. The remaining monosaccharides could largely be assigned to pectic polymers (in total about 42 mol%) with arabinose and galactose being the most important units of rhamnogalacturonan I side chains, and galacturonic acid being a constituent of all pectic polysaccharides. It should be noted, however, that uronic acids are underestimated by using sulfuric acid hydrolysis. Overall, the insoluble dietary fiber monosaccharide composition was very similar to the recently published data of the composition of non-starch polysaccharides from apples after sulfuric acid hydrolysis [25].

The data of extruded samples indicate that insoluble fiber arabinose contents decreased by up to 7% (absolute) with increasing thermo-mechanical treatment, which is in agreement with the data published by Hwang et al. [32]. Also, the portion of galacturonic acid decreased in correlation with an increase in glucose and xylose.

The monosaccharide composition of apple pomace soluble dietary fiber polysaccharides was determined after methanolysis (Table 3). Not surprisingly, the constituents of pectic polysaccharides clearly dominated the soluble fiber fraction. Arabinose was the main monosaccharide (42.0 mol%), followed by galacturonic acid (32.9 mol%) and galactose (8.8 mol%). Similarly to the insoluble dietary fiber polysaccharides, the amount of arabinose decreased by applying thermo-mechanical stress. However, unlike in the case of insoluble fiber, comparably weak thermo-mechanical treatment already had a distinct effect on arabinose portions, which were only slightly decreased with harsher treatment. Galactose and xylose containing polysaccharides appeared to be more stable, as indicated by the growing portions with harsher treatment.

However, these data and additional structural data have to be interpreted keeping in mind two mechanisms of how the compositions of the soluble and insoluble dietary fiber preparations can be altered: 1) solubilization of formerly insoluble polysaccharides, shifting them into the soluble fiber fraction, and 2) actual decomposition of specific structural units in the insoluble or soluble fiber fraction. Also, carbohydrates may be degraded or converted into Maillard products, thus escaping our analyses. As these mechanisms occur simultaneously, definite conclusions are sometimes difficult to draw.

The low–molecular-weight soluble dietary fiber that was produced in the 100-22-700 was mainly composed of arabinose and galactose (35.9% and 18.2%), respectively, and, surprisingly, glucose (41.8%). Xylose (0.9%) and mannose (3.1%) were only minor constituents.

Methylation analysis of the insoluble dietary fiber fraction showed that glucose was mainly a constituent of cellulose (1,4-linked) with lower portions being involved in xyloglucans (1,4,6-linked; 1,4-linked) (Table 4). 1,2-Linked xylopyranose units may have occurred as side chains of xyloglucans. Terminal xylopyranose could be assigned to both xyloglucans and xylans. However, low amounts of 1,4-linked xylopyranose (1.9 mol%) suggested small amounts of xylans as secondary cell wall components only. The identification of mannose after sulfuric acid hydrolysis and low levels of 1,4-linked mannopyranose and 1,4,6-linked mannopyranose demonstrated the existence of (partially branched) mannans in the insoluble fiber fraction. Methylation analysis confirmed that arabinose was mostly integrated in arabinans (1,5-linked) with branches in position *O*-2, *O*-3, or in both positions (1,2,5-, 1,3,5- and 1,2,3,5- linked arabinofuranose units). Branches in position *O*-3 dominated over branches in position *O*-2, as previously demonstrated by Wefers, Flörchinger et al. [25], and doubly substituted arabinose units were quite common. Galactans were mostly unsubstituted (1,4-linked). Methylation analysis data demonstrated the impact of extrusion on insoluble fiber polysaccharides, confirming assumptions made from the analysis of the monosaccharide composition. Changes were most obvious in samples treated at a screw speed of 700 min^−1^: terminal arabinofuranose and 1,3,5-linked arabinofuranose portions were distinctly decreased. Also, doubly substituted arabinan units were decreased using the harshest extrusion conditions. Comparable observations were also made by Schmid et al. [61], who studied thermo-mechanically treated aronia pomace. Galactans, which are often difficult to fully capture using methylation analysis, did not show a clear extrusion affected behavior.

To confirm the methylation analysis data on arabinans and galactans, an arabinan and galactan screening was performed according to the method described by Wefers, Bunzel et al. [45]. Using *endo*-arabinanases and *endo*-galactanases, linear α-(1→5)-linked regions within the arabinan backbone and β-(1→4)-linked regions within the galactan backbone were cleaved, releasing oligomeric units (Table 5, for structures see Figure A1 in the Supporting Information). The results did not fully support methylation analysis data with regard to *O*-3-linked structural units (as mostly reflected by compound A-4a), but also showed a decrease in doubly substituted arabinan structural elements (as reflected by A5a) and generally demonstrated that arabinan complexity was reduced with the application of thermo-mechanical stress. The galactan screening assay confirmed the existence of galactans but did not reveal additional structural data. Xylans, as reflected by 1,4-linked xylopyranose units in the methylation analysis, appeared to be more stable during extrusion and were enriched in the 100-22-700 sample.

Methylation analysis of the soluble dietary fiber fraction confirmed the dominance of the arabinan building blocks among the neutral monosaccharides. Just as seen for the insoluble fiber fraction, branches in position *O*-3 dominated over branches in position *O*-2, and doubly substituted arabinose units were shown to be important structural features in apple pomace soluble fiber. Most distinct effects were seen when the harshest extrusion conditions were applied (100-22-700). Again, the portion of terminal arabinose units was strongly reduced; however, the effect on 1,3,5- (and 1,2,3,5-) linked arabinose units was negligible. Although the changes were minor, xyloglucans may have been enriched in this extruded sample as compared to the raw material. Application of the enzymatic arabinan and galactan profiling assay confirmed the existence of the structural units determined by using methylation analysis (Table A5 and Table A6, Supporting Information) but did not reveal any additional information with respect to the stability of structural units against thermo-mechanical stress.

The degree of esterification of polymer-bound galacturonic acid affects gel formation depending on the gel formation mechanism used. Our data suggest that thermo-mechanical stress reduced the degree of esterification, as clearly demonstrated for the insoluble dietary fiber fraction: the degree of esterification decreased with increasing screw speed from 50% (raw material) to 15% (100-22-700). Comparable behavior was observed for soluble dietary fiber pectic polysaccharides. Modifications with acetates appeared to be more stable than with methyl esters; however, a reduction of the acetylation degree in the extruded samples was observed for the insoluble dietary fiber pectic polysaccharides.

### 3.4. Influence of Process Conditions on Surface and Porosity

**Figure 10:**
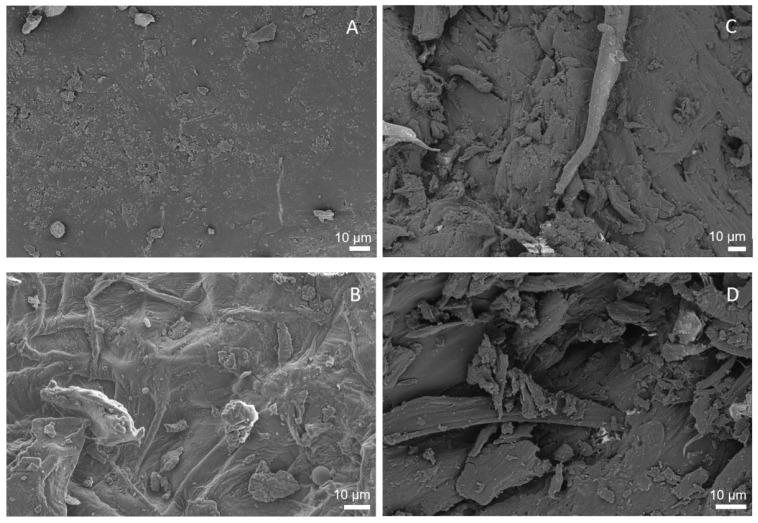
Scanning electron microscope photographs of apple pomace surfaces. (**A**,**B**) show the raw material. (**C**,**D**) show the surface of apple pomace extruded at *T*_barrel_ = 100 °C and a water content of 17%, using a screw speed of 200 min^−1^ (100-17-200).

The surfaces of raw apple pomace (left: A, B) and extruded apple pomace (right: C, D) were visualized by SEM (Zeiss LEO1530). Two representative photographs were selected for both samples and compared in Figure 10.

Figure 10A,B show a rigid closed surface structure of the raw material, whereas the ruptured surface structure of the extruded sample can be seen in Figure 10C,D. Apparently, extrusion disrupted and altered the macroscopic structure of cell walls, resulting in more porous structures.

Therefore, the pore structure of the sieved samples was further analyzed by using a staining technique. Using two different dyes, the pore size distribution and thus the accessible surface area, where dyes could adsorb, could be determined. Direct Yellow 4 (DY 4) populated smaller pores (total accessible surface for molecules: 542 Å^2^, volume: 548 Å^3^), whereas Direct Red 28 (DR 28) populated larger pores (surface: 539 Å^2^, volume: 526 Å^3^) [62].

Compared to measurements of dry powder by BET, staining methods reflected pore structures in the hydrated state, which is relevant to the water absorption and gelling behavior. Table 6 shows the absorbance of the dye DY 4 and DR 28 by the raw material and the extruded samples, which are characterized in detail in Section 3.3.

With decreasing water content during extrusion and increasing screw speed, the sample absorbed more DY 4 dye and less DR 28. Due to their different sizes, it can be concluded that extrusion processing can lead to more small pores, which can be infiltrated by DY 4.

These results suggest that the surface area and porosity are increased by thermo-mechanical treatment during extrusion. Concurrently, water might infiltrate more easily into the cell walls and contribute to the dissolution of the soluble fraction.

## 4. Conclusions

This study shows that valuable residues like apple pomace can be modified and functionalized by extrusion processing. The analysis of the functional properties shows that water solubility increased up to 33%, water absorption up to 22% and paste viscosity up to 20 times for the highest thermo-mechanical treatment (*n* = 700 min^−1^, *m*_H2O_ = 17%) applied. The increase in viscosity was the most prominent modification of the extruded samples. The extent of the thermo-mechanical treatment could be varied significantly by reducing the water content, which was identified to be the main influencing parameter to alter the hydration properties and viscosity of aqueous apple pomace dispersions.

The analysis of the structural components showed changes with increasing thermo-mechanical treatment: a decrease in insoluble dietary fiber as well as an increase in soluble dietary fiber up to low-molecular soluble dietary fiber at maximum screw speed (*n* = 700 min^−1^, *m*_H2O_ = 22%) was observed. More precisely, a decrease in arabinose and also galacturonic acid constituents in the insoluble dietary fiber was measured, indicating solubilization or degradation of previously insoluble pectic polysaccharides during extrusion. The SEM images and Simons’ stain results showed that extrusion not only induced modifications on a molecular level, but also affected macromolecular structures, thus increasing the porosity of the samples, which may explain the increase in WAI. These trends were independent of the particle size of the extruded material, suggesting that the entire material after grinding can be used for applications in foods, e.g., bread, instead of using a specific particle size fraction only.

Nevertheless, to adjust the techno-functional properties according to the desired and specific needs, it is necessary to control the functionalization of apple pomace during the extrusion process more closely. Thus, further studies on the individual and defined effects of both thermal and mechanical stresses on structural changes on a microscopic and molecular level and related techno-functionality are needed.

## Figures and Tables

**Table 1:** Insoluble, soluble, and low-molecular-weight soluble dietary fiber contents (g/100 g dry matter) of raw and extruded apple pomace (mean value ± standard deviation, *n* = 3). For all samples, the barrel temperature was 100 °C.

	Raw Material	100-42-200	100-22-200	100-22-450	100-22-700
Total content ^a^	67.6 ± 1.7 ^A^	60.7 ± 1.8 ^B^	56. 6 ± 2.9 ^C^	60.1 ± 1.7 ^B,C^	63.4 ± 2.1 ^A,B^
Insoluble dietary fiber ^a^	52.8 ± 1.2 ^A^	45.6 ± 1.3 ^B^	43.5 ± 1.9 ^B^	43.3 ± 1.0 ^B^	45.4 ± 0.6 ^B^
Soluble dietary fiber ^a^	12.5 ± 1.2 ^A^	15.1 ± 0.5 ^A^	13.1 ± 1.0 ^B,C^	16.8 ± 0.7 ^A^	16.7 ± 1.3 ^A^
Low-molecular-weight soluble fiber	-	-	-	-	1.3 ± 0.2

^a^ Mean values within a row that are marked with different letters differ significantly (*p* < 0.05).

**Table 2:** Monosaccharide composition (mol%) of insoluble dietary fiber of raw and extruded apple pomace after sulfuric acid hydrolysis (mean value ± range/2, *n* = 2) Fuc: fucose, Rha: rhamnose, Ara: arabinose, Gal: galactose, Glc: glucose, Xyl: xylose, Man: mannose, GalA: galacturonic acid.

	Raw Material	100-42-200	100-22-200	100-22-450	100-22-700
Fuc	1.0 ± 0.01	^a^	^a^	^a^	^a^
Rha	2.3 ± 0.05	2.2 ± 0.2	1.6 ± 0.01	1.6 ± 0.1	1.5 ± 0.1
Ara	17.3 ± 0.8	15.6 ± 0.3	12.9 ± 0.1	10.3 ± 0.2	9.9 ± 0.01
Gal	10.1 ± 0.2	10.7 ± 0.1	11.5 ± 0.4	9.8 ± 0.1	11.0 ± 0.2
Glc	43.4 ± 0.3	47.1 ± 0.5	48.7 ± 0.4	52.9 ± 0.3	53.0 ± 0.2
Xyl	11.0 ± 0.01	11.4 ± 0.03	12.7 ± 0.1	13.7 ± 0.5	13.6 ± 0.3
Man	3.0 ± 0.2	2.8 ± 0.1	3.2 ± 0.4	3.8 ± 0.2	4.1 ± 0.1
GalA	11.9 ± 0.8	10.1 ± 0.7	9.3 ± 0.4	7.8 ± 0.4	6.9 ± 0.1

^a^ Below the limit of quantification.

**Table 3:** Monosaccharide composition (mol%) of soluble dietary fiber of raw and extruded apple pomace after methanolysis (mean value ± range/2, *n* = 2) Fuc: fucose, Rha: rhamnose, Ara: arabinose, Gal: galactose, Glc: glucose, Xyl: xylose, Man: mannose, GlcA: glucuronic acid, GalA: galacturonic acid.

	Raw Material	100-42-200	100-22-200	100-22-450	100-22-700
Fuc	^a^	^a^	^a^	0.7 ± 0.04	0.8 ± 0.01
Rha	4.4 ± 0.2	^-^	^-^	^-^	^-^
Ara	42.0 ± 1.1	34.6 ± 3.1	34.5 ± 0.6	33.0 ± 0.8	33.3 ± 0.6
Gal	8.8± 0.9	11.0 ± 1.4	15.0 ± 0.6	16.1 ± 0.4	19.0 ± 0.7
Glc	4.7 ± 1.4	4.5 ± 0.1	4.4 ± 0.8	3.4 ± 0.3	4.1 ± 0.1
Xyl	1.7 ± 0.3	2.3 ± 2.3	3.6 ± 0.2	3.8 ± 0.6	5.8 ± 0.05
Man	4.7 ± 0.7	1.1 ± 1.1	1.6 ± 0.1	1.1 ± 0.1	1.4 ± 0.1
GalA	32.9 ± 0.4	44.6 ± 2.6	39.4 ± 1.3	40.4 ± 0.7	34.1 ± 1.2
GlcA	0.8 ± 0.05	1.7 ± 0.1	1.6 ± 0.1	1.4 ± 0.1	1.5 ± 0.1

^a^ Below the limit of quantification.

**Table 4:** Glycosidic linkages (partially methylated alditol acetates; mol%) of insoluble dietary fiber of raw and extruded apple pomace (mean value ± range/2, *n* = 2). t: terminal, *p*: pyranose, *f*: furanose, Rha: rhamnose, Ara: arabinose, Gal: galactose, Glc: glucose, Man: mannose, Xyl: xylose.

	Raw Material	100-42-200	100-22-200	100-22-450	100-22-700
1,2-Rha*p*	0.8 ± 0.1	0.4 ± 0.1	0.4 ± 0.1	0.6 ± 0.1	0.8 ± 0.2
1,2,4-Rha*p*	0.4 ± 0.1	0.4 ± 0.1	0.4 ± 0.1	0.4 ± 0.1	0.6 ± 0.3
∑ Rha	1.2 ± 0.2	0.8 ± 0.2	0.8 ± 0.3	1.0 ± 0.2	1.4 ± 0.5
t-Ara*f*	13.5 ± 2.7	8.9 ± 1.0	8.6 ± 1.0	7.1 ± 0.1	8.4 ± 1.2
t-Ara*p*	^a^	1.5 ± 1.1	1.5 ± 1.0	0.5 ± 0.1	1.2 ± 0.2
1,5-Ara*f*/1,4-Ara*p*	8.4 ± 0.04	8.9 ± 1.1	8.6 ± 1.0	9.0 ± 2.1	11.2 ± 2.7
1,3-Ara*f*	^a^	1.2 ± 0.03	1.2 ± 0.03	0.9 ± 0.2	1.6 ± 0.1
1,2,5-Ara*f*	1.2 ± 0.1	1.6 ± 0.4	1.5 ± 0.3	1.3 ± 0.2	1.0 ± 0.1
1,3,5-Ara*f*	4.3 ± 0.3	2.8 ± 0.2	2.7 ± 0.2	1.5 ± 0.2	1.2 ± 0.7
1,2,3,5-Ara*f*	6.6 ± 0.5	8.8 ± 1.1	8.5 ± 1.0	5.1 ± 1.6	4.1 ± 0.1
∑ Ara	34.0 ± 3.6	33.6 ± 4.8	32.6 ± 4.7	25.5 ± 4.4	28.6 ± 5.0
t-Gal*p*	2.9 ± 0.3	2.8 ± 0.3	2.7 ± 0.3	2.7 ± 0.03	4.1 ± 0.3
1,4-Gal*p*	3.1 ± 0.1	2.6 ± 0.3	2.5 ± 0.3	2.4 ± 0.1	3.9 ± 0.1
1,6-Gal*p*	0.3 ± 0.1	0.3 ± 0.03	0.3 ± 0.03	0.6 ± 0.05	1.2 ± 0.01
∑ Gal	6.3 ± 0.6	5.7 ± 0.6	5.5 ± 0.6	5.7 ± 0.2	9.1 ± 0.4
t-Glc*p*	1.2 ± 0.04	1.3 ± 0.3	1.3 ± 0.3	1.7 ± 0.2	1.8 ± 0.4
1,4-Glc*p*	35.8 ± 4.2	33.8 ± 0.6	32.8 ± 0.4	36.8 ± 1.6	24.9 ± 1.7
1,4,6-Glc*p*	5.7 ± 1.4	8.2 ± 1.0	8.6 ± 1.0	9.9 ± 0.1	5.6 ± 2.4
∑ Glc	42.7 ± 5.7	42.1 ± 1.6	42.7 ± 1.7	48.4 ± 1.9	32.3 ± 4.5
1,4-Man*p*	2.9 ± 0.8	2.9 ± 0.4	2.9 ± 0.4	2.9 ± 0.1	4.5 ± 0.1
1,4,6-Man*p*	0.5 ± 0.2	^a^	^a^	^a^	^a^
∑ Man	3.4 ± 1.0	2.9 ± 0.4	2.9 ± 0.4	2.9 ± 0.1	4.5 ± 0.1
t-Xyl*p*	7.8 ± 0.6	9.2 ± 0.7	8.9 ± 0.7	8.7 ± 0.5	12.7 ± 0.3
1,2-Xyl*p* ^b^	2.7 ± 0.5	2.3 ± 0.2	3.0 ± 0.1	3.8 ± 0.4	6.0 ± 0.1
1,4-Xyl*p* ^b^	1.9 ± 0.2	2.0 ± 0.03	3.5 ± 0.4	4.0 ± 0.3	5.4 ± 0.7
∑ Xyl	12.4 ± 1.4	13.5 ± 0.9	15.4 ± 1.2	16.6 ± 1.2	24.0 ± 1.2

^a^ Not detected; ^b^ Coeluting, determined from the area ratio of the characteristic fragment ion peaks. 1,2-Xyl*p*: *m*/*z* 117, 1,4-Xyl*p*: *m*/*z* 118.

**Table 5:** Arabinan and galactan oligosaccharide composition (mol%) after incubation of insoluble dietary fiber of raw and extruded apple pomace with *endo*-arabinanase and *endo*-galactanase (mean value ± range/2, *n* = 2).

Compound	Raw Material	100-42-200	100-22-200	100-22-450	100-22-700
Arabinan oligosaccharide					
A-2a	84.0 ± 0.4	84.2 ± 0.3	87.6 ± 0.05	83.5 ± 0.8	83.8 ± 0.1
A-4a	7.2 ± 0.4	10.5 ± 0.05	10.2 ± 0.1	11.9 ± 0.8	11.1 ± 0.2
A-4b	-	1.7 ± 0.1	1.1 ± 0.01	2.2 ± 0.1	2.0 ± 0.3
A-5a	4.1 ± 0.02	1.3 ± 0.1	1.0 ± 0.1	0.7 ± 0.1	1.1 ± 0.1
A-5b	2.1 ± 0.1	2.4 ± 0.1	-	1.7 ± 0.1	2.0 ± 0.03
A-5c	0.7 ± 0.1	-	-	^a^	^a^
A-7a	1.9 ± 0.1	-	-	-	-
Galactan oligosaccharide					
G-2a	98.3 ± 0.3	100.0	100.0	100.0	100.0
G-2b	-	-	^a^	^a^	-
G-2c	1.7 ± 0.3	^a^	^a^	^a^	-

^a^ Below the limit of quantification.

**Table 6:** Absorption of DY 4 and DR 28 of raw material and various extruded apple pomace samples for a barrel temperature of 100 °C.

	Absorbed Direct Yellow 4 [mg_dye_·g_sample_^−1^]	Absorbed Direct Red 28 [mg_dye_·g_sample_^−1^]
raw material	5.541 ± 0.013	9.827 ± 0.018
100-42-200	6.208 ± 0.009	9.815 ± 0.011
100-22-200	6.749 ± 0.007	9.732 ± 0.010
100-22-450	6.712 ± 0.009	8.809 ± 0.006
100-22-700	6.876 ± 0.014	8.103 ± 0.003

**Figure A1:**
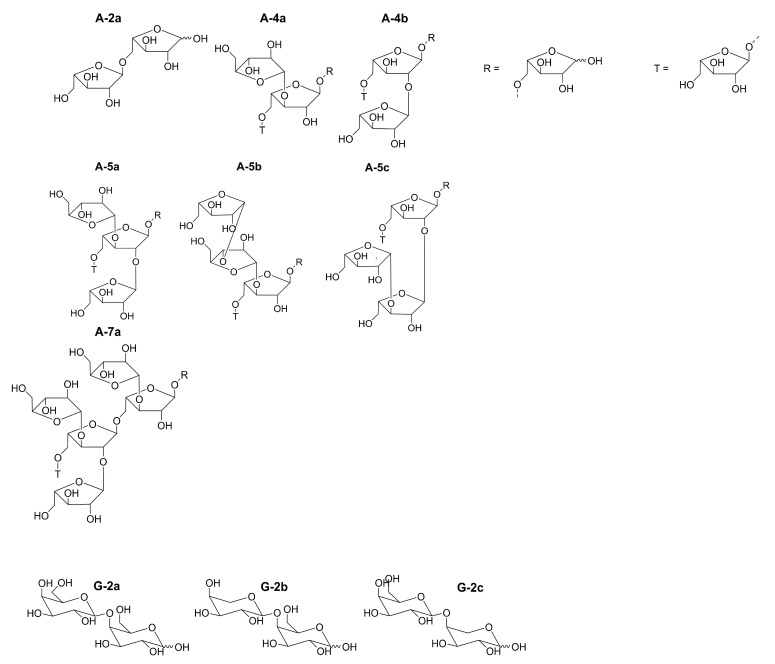
Structures of arabinan and galactan oligosaccharides of raw and extruded apple pomace dietary fiber after enzymatic cleavage by *endo*-arabinanase and *endo*-galactanase. R: reducing end, T: terminal end.

## Appendix A

### Appendix A.1. Supporting Methods

Protein content. Sample material (100 mg) was digested by using the Kjedahl approach, and ammonia detection was performed using an ammonia electrode [41].

Fat content. Sample material (5–10 g) was weighed into a fat-free extraction tube and extracted in a Soxleth apparatus for 1 h 22 min using petroleum ether as solvent.

Ash content. Sample material (1 g) was weighed into a crucible. After incineration at 500 °C for 4 h, the ash content was determined gravimetrically.

### Appendix A.2. Supporting Tables

**Table A1:** Monosaccharide composition (mol%) of the polysaccharides of the sieved and non-sieved fraction of apple pomace (raw and extruded) after sulfuric acid hydrolysis and methanolysis (mean value ± range/2, *n* = 2). Ara: arabinose, Gal: galactose, Glc: glucose, Xyl: xylose, Man: mannose, GalA: galacturonic acid, Rha: rhamnose, GlcA: glucuronic acid.

Sulfuric Acid Hydrolysis										
	Raw Material		100-42-200		100-22-200		100-22-450		100-22-700	
	Sieved	Non-sieved	Sieved	Non-sieved	Sieved	Non-sieved	Sieved	Non-sieved	Sieved	Non-sieved
Ara	16.4 ± 0.3	15.1 ± 0.3	16.1 ± 0.05	16.1 ± 0.03	15.8 ± 0.2	16.1 ± 0.1	16.4 ± 0.1	16.3 ± 0.1	16.1 ± 0.4	15.9 ± 0.2
Gal	10.1 ± 0.4	9.4 ± 0.5	10.7 ± 0.4	9.4 ± 0.5	10.7 ± 0.1	9.9 ± 0.02	14.4 ± 0.2	13.1 ± 0.3	15.0 ± 0.2	13.6 ± 1.1
Glc	43.1 ± 1.1	45.4 ± 0.6	44.0 ± 0.1	42.5 ± 0.2	43.5 ± 0.4	42.0 ± 0.6	44.1 ± 0.1	42.5 ± 0.2	43.8 ± 0.01	44.5 ± 1.6
Xyl	7.1 ± 0.2	8.0 ± 0.3	7.0 ± 0.2	8.0 ± 0.3	7.0 ± 0.06	8.1 ± 0.5	7.8 ± 0.1	8.0 ± 0.2	7.4 ± 0.1	8.7 ± 0.1
Man	2.5 ± 0.1	2.5 ± 0.2	2.6 ± 0.1	2.1 ± 0.04	2.2 ± 0.1	2.1 ± 0.01	2.3 ± 0.01	2.2 ± 0.2	2.3 ± 0.1	2.5 ± 0.2
GalA	20.8 ± 0.2	19.6 ± 0.8	19.6 ± 0.8	21.2 ± 0.2	20.9 ± 0.03	22.3 ± 0.2	18.6 ± 0.03	21.1 ± 0.3	18.3 ± 0.9	16.2 ± 1.9
**Methanolysis**										
Rha	2.1 ± 0.1	2.3 ± 0.05	2.0 ± 0.2	1.9 ± 0.05	1.6 ± 0.3	1.7 ± 0.001	1.6 ± 0.01	1.5 ± 0.2	1.4 ± 0.3	1.4 ± 0.3
Ara	23.2 ± 0.5	23.8 ± 0.1	25.6 ± 0.6	22.5 ± 0.1	22.3 ± 0.9	22.1 ± 0.6	22.7 ± 0.9	22.0 ± 0.2	23.1 ± 1.5	24.3 ± 2.0
Gal	13.5 ± 0.4	14.2 ± 0.2	15.0 ± 0.3	13.1 ± 0.3	13.0 ± 0.2	13.3 ± 0.3	14.4 ± 0.2	13.1 ± 0.3	15.0 ± 0.2	13.6 ± 1.1
Gluc	12.9 ± 0.5	13.7 ± 0.5	14.4 ± 0.3	15.1 ± 0.2	14.6 ± 0.3	15.4 ± 0.05	15.7 ± 0.04	15.8 ± 0.2	14.5 ± 0.2	14.0 ± 0.1
Xyl	11.5 ± 0.3	12.8 ± 0.2	11.9 ± 0.6	12.5 ± 0.1	13.4 ± 0.3	13.5 ± 0.004	11.7 ± 0.4	11.8 ± 0.4	11.9 ± 0.4	10.9 ± 0.5
Man	1.4 ± 0.1	1.2 ± 0.01	1.3 ± 0.003	1.5 ± 0.1	1.7 ± 0.2	1.7 ± 0.1	1.6 ± 0.1	1.5 ± 0.3	1.5 ± 0.1	1.3 ± 0.3
GalA	35.0 ± 0.8	31.5 ± 0.7	29.9 ± 0.8	32.9 ± 0.6	32.8 ± 0.5	31.9 ± 0.2	31.9 ± 0.7	33.8 ± 0.7	31.9 ± 1.4	33.7 ± 2.4
GlcA	0.4 ± 0.01	0.5 ± 0.02		0.5 ± 0.03	0.6 ± 0.1	0.5 ± 0.01	0.6 ± 0.04	0.4 ± 0.1	0.6 ± 0.1	0.7 ± 0.02

**Table A2:** Protein, fat, and ash contents (g/100 g dry matter) of raw and extruded apple pomace (mean value ± standard deviation, *n* = 3).

	Raw Material	100-42-200	100-22-200	100-22-450	100-22-700
Protein content ^a^	5.0 ± 0.5 ^B^	5.6 ± 0.4 ^B,A^	5.5 ± 0.4 ^B^	5.3 ± 0.2 ^B^	6.6 ± 0.4 ^A^
Fat content ^a^	1.6 ± 0.02 ^A^	1.6 ± 0.1 ^A^	2.1 ± 0.2 ^A,B^	2.3 ± 0.4 ^B^	2.0 ± 0.2 ^A,B^
Ash content ^a^	1.4 ± 0.2 ^A^	1.1 ± 0.1 ^A,B^	1.0 ± 0.1 ^A,B^	1.0 ± 0.1 ^B^	1.0 ± 0.2 ^A,B^

^a^ Mean values within a row that are marked with different letters differ significantly (*p* < 0.05).

**Table A3:** Contents of free mono- and disaccharides (g/100 g dry matter) of raw and extruded apple pomace (mean value ± standard deviation, *n* = 3).

	Raw Material	100-42-200	100-22-200	100-22-450	100-22-700
Glucose ^a^	2.8 ± 0.3 ^A^	3.5 ± 0.3 ^A^	2.9 ± 0.1 ^A^	3.1 ± 0.1 ^A^	3.0 ± 0.5 ^A^
Fructose ^a^	8.8 ± 0.3 ^A^	5.1 ± 0.5 ^B^	4.1 ± 0.3 ^C,D^	4.3 ± 0.1 ^B,C^	3.4 ± 0.1 ^D^
Sucrose ^a^	2.3 ± 0.4 ^B,C^	4.4 ± 0.2 ^A^	2.8 ± 0.2 ^B^	1.9 ± 0.2 ^C^	0.7 ± 0.5 ^D^
Maltose	1.4 ± 0.3	^b^	^b^	^b^	^b^

^a^ Mean values within a row that are marked with different letters differ significantly (*p* < 0.05); ^b^ Below the limit of quantification.

**Table A4:** Starch contents (g/100 g dry matter) of raw and extruded apple pomace (mean value ± standard deviation, *n* = 3).

	Raw Material	100-42-200	100-22-200	100-22-450	100-22-700
Starch content ^a^	11.6 ± 0.7 ^A^	9.2 ± 0.8 ^B^	8.8 ± 0.6 ^B^	8.3 ± 0.6 ^B^	8.5 ± 0.8 ^B^

^a^ Mean values within a row that are marked with different letters differ significantly (*p* < 0.05).

**Table A5:** Glycosidic linkages (partially methylated alditol acetates; mol%) of soluble dietary fiber of raw and extruded apple pomace (mean value ± range/2, *n* = 2). t: terminal, *p*: pyranose, *f*: furanose, Rha: rhamnose, Ara: arabinose, Gal: galactose, Glc: glucose, Man: mannose, Xyl: xylose.

	Raw Material	100-40-200	100-20-200	100-20-450	100-20-700
1,2-Rha*p*	^a^	1.4 ± 0.03	1.4 ± 0.03	1.2 ± 0.6	1.0 ± 0.5
1,2,4-Rha*p*	0.9 ± 0.02	0.9 ± 0.03	0.9 ± 0.03	0.9 ± 0.4	1.2 ± 0.01
∑ Rha	0.9 ± 0.02	2.3 ± 0.1	2.3 ± 0.1	2.1 ± 1.0	2.2 ± 0.5
t-Ara*f*	33.3 ± 1.2	25.7 ± 0.6	25.4 ± 0.6	24.5 ± 1.9	20.8 ± 0.1
t-Ara*p*	^a^	0.8 ± 0.01	0.8 ± 0.1	0.9 ± 0.3	0.9 ± 0.01
1,5-Ara*f*/1,4-Ara*p*	21.1 ± 0.4	19.9 ± 0.4	19.7 ± 0.4	21.2 ± 0.8	18.3 ± 0.5
1,3-Ara*f*	^a^	3.3 ± 0.1	3.3 ± 0.1	4.3 ± 0.3	2.4 ± 0.9
1,2,5-Ara*f*	1.3 ± 0.1	2.2 ± 0.1	2.2 ± 0.2	2.3 ± 0.5	2.1 ± 0.1
1,3,5-Ara*f*	8.2 ± 0.5	8.2 ± 0.2	8.1 ± 0.2	9.5 ± 0.2	7.9 ± 0.4
1,2,3,5-Ara*f*	9.2 ± 3.0	11.8 ± 0.2	11.6 ± 0.2	12.8 ± 0.9	7.6 ± 1.0
∑ Ara	73,1 ± 4.0	71.9 ± 1.4	71.2 ± 1.5	75.5 ± 4.9	60.1 ± 3.0
t-Gal*p*	1.1 ± 0.1	2.5 ± 0.04	2.5 ± 0.04	2.1 ± 0.3	3.4 ± 0.03
1,4-Gal*p*	2.3 ± 0.3	6.2 ± 1.7	6.1 ± 1.7	5.1 ± 0.8	7.7 ± 1.3
1,6-Gal*p*	^a^	^a^	0.3 ± 0.04	^a^	^a^
∑ Gal	3.3 ± 0.5	8.7 ± 1.7	8.9 ± 1.8	7.3 ± 1.2	11.0 ± 1.4
t-Glc*p*	2.4 ± 0.2	0.8 ± 0.01	0.8 ± 0.01	0.8 ± 0.2	0.7 ± 0.1
1,4-Glc*p*	12.4 ± 1.9	7.8 ± 0.2	7.7 ± 0.2	5.4 ± 1.2	10.8 ± 1.2
1,4,6-Glc*p*	1.2 ± 0.1	2.7 ± 0.01	2.7 ± 0.01	2.8 ± 0.01	4.8 ± 0.1
∑ Glc	15.9 ± 2.2	11.3 ± 0.2	11.2 ± 0.2	9.0 ± 1.4	16.3 ± 1.3
1,4-Man*p*	1.4 ± 0.1	1.0 ± 0.1	1.0 ± 0.1	0.8 ± 0.2	1.6 ± 0.03
∑ Man	1.4 ± 0.1	1.0 ± 0.2	1.0 ± 0.1	0.8 ± 0.2	1.6 ± 0.03
t-Xyl*p*	3.5 ± 0.2	3.6 ± 0.1	3.5 ± 0.1	4.1 ± 0.1	5.7 ± 0.4
1,2-Xyl*p* ^b^	0.8 ± 0.2	0.7 ± 0.02	1.2 ± 0.02	0.5 ± 0.03	1.4 ± 0.05
1,4-Xyl*p* ^b^	1.0 ± 0.1	0.5 ± 0.03	0.7 ± 0.01	0.7 ± 0.003	1.7 ± 0.5
∑ Xyl	5.4 ± 0.5	4.8 ± 0.1	5.4 ± 0.1	5.3 ± 0.1	8.9 ± 1.0

^a^ Not detected; ^b^ Coeluting, determined from the area ratio of the characteristic fragment ion peaks. 1,2-Xyl*p*: *m*/*z* 117, 1,4-Xyl*p*: *m*/*z* 118.

**Table A6:** Arabinan and galactan oligosaccharide composition (mol%) after incubation of soluble dietary fiber of raw and extruded apple pomace with *endo*-arabinanase and *endo*-galactanase (mean value ± range/2, *n* = 2).

	Raw Material	100-40-200	100-20-200	100-20-450	100-20-700
Arabinan oligosaccharide					
A-2a	84.7 ± 0.01	79.0 ± 1.4	77.0 ± 2.9	78.1 ± 2.4	78.8 ± 0.2
A-4a	6.9 ± 0.1	0.01	14.0 ± 2.5	15.1 ± 0.4	14.7 ± 0.2
A-4b	-	3.1 ± 0.03	3.4 ± 0.8	3.6 ± 0.1	3.3 ± 0.04
A-5a	3.4 ± 0.1	4.9 ± 1.4	3.3 ± 1.2	3.1 ± 1.7	2.1 ± 0.1
A-5b	2.6 ± 0.1	^a^	2.3 ± 0.9	^a^	1.1 ± 0.1
A-5c	0.7 ± 0.0004	^a^	^a^	^a^	-
A-7a	1.7 ± 0.1	-	-	-	-
Galactan oligosaccharide					
G-2a	100.0	100.0	99.0 ± 0.1	100.0	100.0
G-2b	-	^a^		-	-
G-2c	-	-	1.0 ± 0.1	-	-

^a^ Below the limit of quantification.
